# Immediate effect of non-invasive auricular acupoint stimulation on the performance and meridian activities of archery athletes: A protocol for randomized controlled trial

**DOI:** 10.1097/MD.0000000000024753

**Published:** 2021-02-26

**Authors:** Yi-Hsun Tsai, Szu-Ying Wu, Wen-Long Hu, Yun-Ru Lai, Yu Tsao, Ke-Tien Yen, Cheng-Hsien Lin, Chun-En Aurea Kuo

**Affiliations:** a Department of Chinese Medicine, Kaohsiung Chang Gung Memorial Hospital and Chang Gung University College of Medicine, Kaohsiung; b Department of Nursing, Meiho University, Pingtung; c Department of Sports Medicine, Kaohsiung Medical University; d College of Medicine, Kaohsiung Medical University, Kaohsiung; e Fooyin University College of Nursing; f Department of Biological Science, National Sun Yat-Sen University; g Department of Neurology Chang Gung Memorial Hospital, Chang Gung University College of Medicine; h Department of Leisure and Sports Management, Cheng Shiu University; i College of Management, National Kaohsiung University of Science and Technology, Kaohsiung; j Department of Sports Training Science-Athletics, National Taiwan Sport University, Taoyuan, Taiwan.

**Keywords:** acupressure, archery, autonomic function, precision sports

## Abstract

**Background:**

Archery has existed in human history for millenniums. Being a unique exercise and precision sport, the keys to performance are emotional control, attention, and concentration rather than explosive force, muscle power, and endurance. During the execution of archery, attention is the key to performance in elite players, especially in the initial period while drawing the bow. Auricular acupoint stimulation is one of the therapeutic methods of traditional Chinese medicine and has been reported for its use in amplifying the anesthesia effect, weight reduction, cessation of substance abuse, and autonomic nervous modulation.

**Methods:**

The study will recruit archery players in school teams among junior and senior high schools and colleges. The subjects will be randomly assigned to the ear and sham acupressure groups. This is a randomized controlled trial with crossover design. The outcome measures will be obtained, including the meridian activities and balance index with Ryodoraku device, the movement stability with WIMU tracking system, the continuous heart rate record, and the scores of the 2 sections as the performance. The subjects will rate their attention and fatigue levels through self-reported questionnaires.

**Objectives:**

This study aims to investigate the immediate effect of non-invasive auricular acupoint stimulation on the performance and meridian activities of archery athletes.

**Trial registration:**

ClinicalTrials.gov Identifier: NCT04637607.

## 1 Introduction

Archery is a sport of using a bow to shoot arrows to a designated target with set distance and direction. It was first recorded as a sport in the ancient Egyptian and Chinese histories and is one of the oldest sports that is still practiced. Archery first appeared in the Olympic games in 1900, and has become a regular competition since 1972. Modern competitive archery games include target, field, Clout, crossbow, and flight archeries, etc. Modern popular bow types include compound, recurve, and barebow. Because of different setting and shooting methods, competitions of different bow types are held separately. Since there was no definite influence on performance in different weight classes, archers are divided into groups by different bow types and age according to the rules established by the World Archery Federation (formerly known as FITA from the Fédération Internationale de Tir à l’Arc).

Most sports performances are affected by power, muscular strength, and endurance. However, concentration, emotional control, attention, and stability are more important in precision sports such as archery, shooting, and golf.^[1,2]^ Archers must be stable, focused, and relaxed while performing without interference and fluctuating emotions. Some researchers pointed out that the mental status just before performing sports is the key to performance in precision sports.^[3,4]^ However, concentration and attention are easily disturbed during tournaments.^[5]^ The ability to concentrate from few seconds before action till launching is especially important to archers pursuing excellence.

The correlation between attention and heart rate variability (HRV) was first illustrated by the intake-reject hypothesis proposed by John I. Lacey. The hypothesis mentioned that the acceptance of external environmental stimulation or information is related to decreased heart rate, whereas its rejection is related to increased heart rate. There are multiple researches about precision sports and decreased heart rate. Hatfield et al^[3]^ found that during the aiming period of elite rifle marksmen, decreased heart rate was detected prior to trigger pull compared with baseline. Landers et al^[6]^ monitored heart rate activity of 11 right-handed adult archers during a 15-week beginning archery class. After 14 weeks of training, the archers showed improved performance accompanied with significant heart rate deceleration in the few seconds prior to arrow release compared with their pretrained conditions. Carrillo et al^[7]^ compared archery performance and autonomic nervous system (ANS) modulation by changes in HRV between 7 novices and 10 experienced adolescent archers. Compared with novices, experienced archers revealed a higher square root of the mean of squared differences between successive R-R intervals and a higher percentage of successive normal-to-normal intervals >50 ms. In other words, experienced adolescent archers presented a higher HRV, which may demonstrate a better regulation of parasympathetic nervous system. The activity of ANS plays an important role in archery. The activation of sympathetic nervous system not only increases heart rate, but diminishes stability and performance. Therefore, to activate parasympathetic nervous system well is essential to archers seeking optimal performance.

Auricular acupoint stimulation is one of the therapeutic methods in traditional Chinese medicine and is widely applied in treating various diseases. Previous literatures had concluded the use of auricular acupoint stimulation in amplifying the anesthesia effect,^[8]^ weight reduction,^[9]^ and the cessation of substance abuse.^[10]^ Auricular acupoints could be stimulated with invasive metal auricular needles or non-invasive stimuli, including magnetic beads and Vaccaria seeds.

Hsu et al^[11]^ conducted a research to evaluate HRV, pulse rate variability, and electroencephalogram in 10 healthy adults with electroencephalogram, electrocardiogram, and blood-pressure-pulse recorder after 10 minutes of acupuncture over Sishencong scalp acupoint and Shen-men auricular acupoint separately. Decreased heart rate, pulse rate, and blood pressure were shown after stimulation on either scalp or auricular acupoints compared with control. In frequency domain analysis after fast Fourier transform, the high and low frequency powers of HRV were respectively increased and decreased, which indicated that parasympathetic nervous system was activated and sympathetic nervous system was inhibited. Conclusively, acupuncture over either Sishencong scalp acupoint or Shen-men auricular acupoint might lower the heart rate and activate the parasympathetic nerves, and that is the exact principle to advance archery performance as mentioned above. By applying such effect, we could alleviate the negative effect caused by pressure during competition and improve concentration and stabilization, the necessary characters for archery. However, no previous literature has investigated the effect of acupuncture or acupressure on the autonomic nervous condition or meridian characteristics of archery athletes or their performance.

Combining the modern electrophysiology of nervous system and the meridian theory in traditional Chinese medicine, this randomized controlled study aims to investigate the immediate effect of non-invasive and sham auricular acupoint stimulation on the meridian activities through measuring Ryodoraku and observing whether auricular acupoints could regulate ANS condition, meridian activities, and thus affect performance in archery athletes.

## 2 Methods

### 2.1 Ethics approval

The study was approved by the human ethics committee of Chang Gung Medical Foundation institutional review board (IRB No. 201900969B0C501). The protocol has been registered at ClinicalTrials.gov (identifier: NCT04637607, latest version on January 6, 2021). All participants will provide their written informed consent. Personal information about participants will be collected, shared, and maintained in an independent closet to protect confidentiality before, during, and after the trial. Therefore, the participants will be presented as serial numbers and the conversion list will be stored appropriately in a locked closet. The principal investigator will have access to the final trial dataset.

### 2.2 Study design

**Figure 1:**
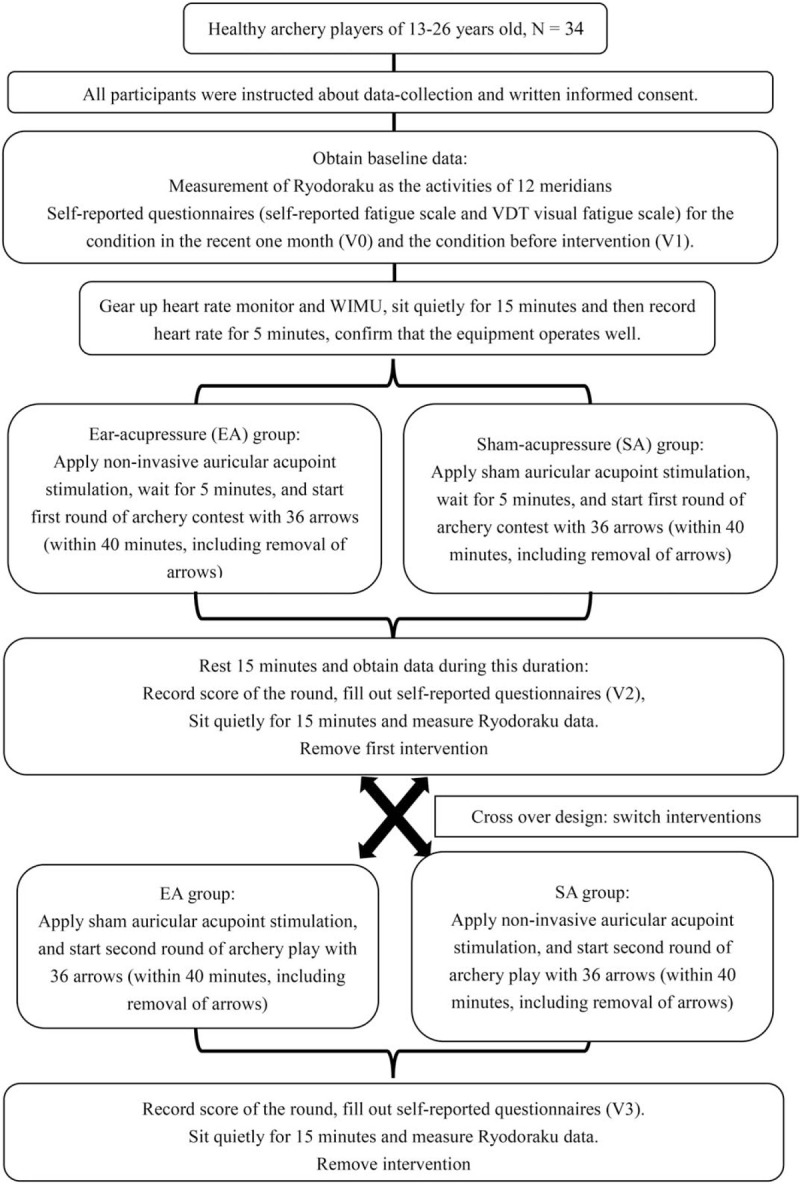
Flow diagram of the experimental design. VDT = visual display terminal.

The study was started in October 2019 and planned to complete by December 2020. This is a crossover randomized controlled trial (Fig. 1). Each participant will be tested with 2 different interventions respectively. The participants will be randomized into ear-acupressure (EA) group (n = 17, anticipated) or sham-acupressure (SA) group (n = 17, anticipated), according to their order of appearance on the measuring day. Participants appearing in the odds order (1st, 3rd, 5th, etc) will be allocated to EA group whereas those appearing in the even order (2nd, 4th, 6th, etc) will be allocated to SA group. The trial participants and outcome assessors will be blinded after assignment to interventions using labels 1 and 2 for the 2 groups. The 2 interventions will be administered on the same day during 2 consecutive rounds of archery contest. The measurement will be performed at the archery ranges where participants used to practice archery.

### 2.3 Participants

Volunteered participants will be recruited from school teams among junior and senior high schools, and colleges in Kaohsiung, Taiwan through poster announcements in schools with archery club or school team and through internet. The investigator anticipates to enroll 34 participants, 17 each on EA and SA groups. Basic information including training duration of archery (1–3, 3–5, 6–10, and >10 years) and medical history will be collected. Inclusion criteria are healthy archers between 13 and 26 years old, who had been training for >1 year. Exclusion criteria include history of psychiatric disorders or cardiovascular diseases and previous surgical history of dominant hand. Those who are unwilling to provide informed consent will also be excluded. The study procedures and possible risks will be well explained to all the participants.

### 2.4 Sample size

26 participants were estimated to be needed based on repeated-measures analysis of variance with an effect size of 0.48, a significance level (α) of 0.05, and a desired power (1–β) of 0.80. The effect size was calculated from previous literature with G∗power, version 3.1.9.7 (Faul et al, 2020).^[12]^ In the study conducted by Molassiotis et al,^[13]^ the mean (standard deviation) of general fatigue scores at the baseline before the treatment were 16.6 (2.7) at the acupressure group, and 17.8 (2.5) at the sham acupressure group, and the mean (standard deviation) of general fatigue scores after completing the treatment were 14 (2.4) at the acupressure group, and 16.9 (3.0) at the sham acupressure group. Taking the potential 20% drop-outs into account, a total of 34 participants will be recruited.

### 2.5 Intervention

Ear acupressure: Vaccaria seeds are the mature seeds of *Vaccaria segetalis (Neck.) Garcke*, also known as cowherb, and wang-bu-lui-xing in Chinese. It is usually spherical in shape and about 1.5 to 2 mm in size. Vaccaria seeds will be attached by 3 M tape over auricular heart, liver, shen-men, and kidney points, under the application of the Chinese medicine physician who has >7 years of clinical service. Five minutes after administration, participants will start shooting a round of play with 36 arrows (within 40 minutes, including removal of arrows).

Sham acupressure: Same size of 3 M tape as in ear acupressure with a marked black point that looks similar to Vaccaria seed, will be attached over auricular heart, liver, shen-men, and kidney points. Five minutes after administration, participants will start shooting a round of play with 36 arrows (within 40 minutes, including removal of arrows).

Immediately after first round of play, the self-reported questionnaire will be filled out by the participants (V2). After sitting quietly for 15 minutes, the participants will receive Ryodoraku measurement. The Vacciria seeds/sham 3 M tape will be removed and switched to the other intervention (crossover) after the assessments are completed. Archers should rest for 15 minutes (including the time of obtaining written questionnaire and Ryodoraku measurement) between 2 rounds of play according to World Archery Federation rules. Next, the participants will start shooting the second round of play. Immediately after second round of play, the self-reported questionnaire will be filled out by the participants (V3). After sitting quietly for 15 minutes, the Ryodoraku measurement will be performed. Participants could cease the experiment if unbearable discomfort is experienced or unstable vital condition is observed during intervention, or under any personal request to stop the process.

### 2.6 Outcome measurements

#### 2.6.1 Primary outcome measurements

##### 2.6.1.1 The change of meridian activities

**Figure 2:**
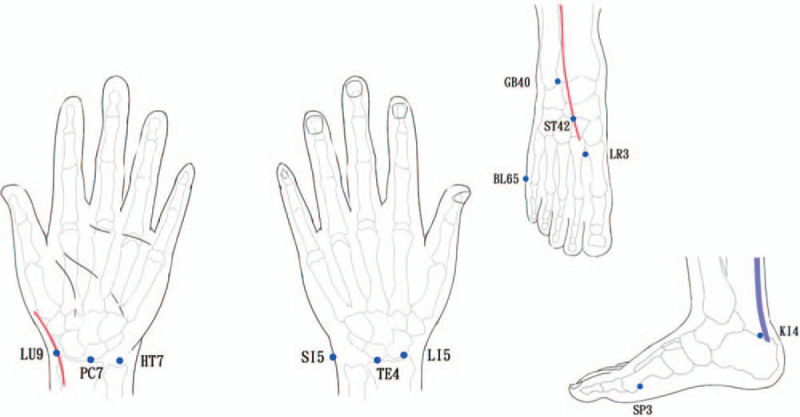
Measured Ryodotens. Each Ryodoten represents the activity of respective meridian and the balance index will be calculated.

The meridian activities and balance index will be measured and calculated with Ryodoraku device (Meridian Energy Analysis Device, MEAD, medpex, Taiwan). It will be measured 3 times: before intervention (baseline) and 1 and 2 hours after intervention. The meridian activities will be shown by numbers calculated by the skin electric resistance. The higher the number, the higher is the meridian activity, and vice versa. Before the Ryodoraku measurement, the participants need to take off their shoes and socks, remove all metal accessories, cellphone, medication, electronic devices on their body, clean their hands and feet, and lie down on the plastic bed. During the measurement, the participant must not contact any metal obstacle and the investigator will not touch the participant. The 12 measured Ryodotens will be as follows (Fig. 2): Taiyuan (LU9) on the Lung meridian, Daling (PC7) on the Pericardium meridian, Shenmen (HT7) on the Heart meridian, Yanggu (SI5) on the Small Intestine meridian, Yangchi (TE4) on the Tripple Energizer meridian, Yangxi (LI5) on the Large Intestine meridian, Taibai (SP3) on the Spleen meridian, Taichong (LR3) on the Liver meridian, Dazhong (KI4) on the Kidney meridian, Shugu (BL65) on the Bladder meridian, Qiuxu (GB40) on the Gallbladder meridian, and Chongyang (ST42) on the Stomach meridian. Each first detected data will be viewed as the activity of that Ryodoten.

##### 2.6.1.2 The change of HRV

The change of HRV will be analyzed by continuous heart rate recording with the HRM-Dual heart rate monitor (Garmin, Schaffhausen, Switzerland. https://buy.garmin.com/en-AU/AU/p/649059/pn/010-12883-00). It provides continuous heart rate record, transmits real-time heart rate data over ANT+ connectivity, and features a soft strap that is comfortable and adjustable. The heart rate will be recorded during the whole process for 2 and 1/2 hours so that the change in heart rate and HRV could be analyzed.

##### 2.6.1.3 The change of checklist individual strength

The self-reported attention and fatigue questionnaire contains 30 items that are composed of subjective fatigue experience, concentration, motivation, and physical activity levels. Each item will be ranked from 1 to 5. Higher scores indicate higher distraction and fatigue levels. The questionnaires will be filled out 3 times: before intervention (baseline) and 1 and 2 hours after intervention.

##### 2.6.1.4 The change of visual display terminals (VDTs) visual syndrome

The VDTs fatigue scale is a self-reported attention and fatigue questionnaire that focuses on visual syndrome and associated symptoms of long-time visual usage during work. VDTs fatigue scale has been applied and standardized by Japanese Ministry of Health, Labor, and Welfare survey results, 2004. VDTs fatigue scale is composed of 13 items. Each item ranks from 1 to 5. Higher scores indicate higher distraction and fatigue levels. The questionnaires will be filled out 3 times: before intervention (baseline) and 1 and 2 hours after intervention. This scale has been chosen as the method of evaluation for attention and visual fatigue due to the similar character of highly visual demand in archers and VDT workers.

#### 2.6.2 Secondary outcome measures:

##### 2.6.2.1 The change of motion stability

The change of motion will be recorded and analyzed by the WIMU PRO (RealTrack Systems, Almería, Spain. http://www.realtracksystems.com/wimu-pro/) throughout the whole process for 2 and 1/2 hours. WIMU is a tracking system with GPS/GNSS, 3D accelerometer, 3D gyroscope, 3D magnetometer, and WIFI 802.11 B/G/N, bluetooth 4.1, ANT+, and BLE.

### 2.7 Statistical analysis

All data will be presented as mean ± standard deviation. Chi-square test or Fishers exact test will be applied to compare all patient characteristics and variables at baseline. Repeated measures analysis of variance will be used for comparison of the outcomes between ear and sham acupressures. Statistical significance will be considered at a *P*-value of <.05. All statistical analyses will be performed with SPSS software for Windows, version 25 (Statistics 25, SPSS, IBM Corp., Chicago, IL).

### 2.8 Data monitoring

The institutional review board and Kaohsiung Chang Gung Memorial Hospital's statisticians will be a part of the data monitoring and auditing committee in this study. It is independent from the sponsor and competing interests. Any outcome data will also be collected for participants who discontinue or deviate from intervention protocols. Two study assistance will range checks for data values. The rare but possible side effects of ear acupressure are dizziness, pain, gastrointestinal discomfort, and skin irritation.^[14]^ The small electric flow during Ryodoraku measurement has no influence on human body, and hence will not induce obvious side effect or danger. If adverse events are found during the trial, data and detailed condition will be recorded and analyzed.

## 3 Discussion

The ANS is a control system of human body that regulate physiological activities and internal organ functions to face the fight-or-flight response. It is delicate, complicated, and it involves body movements and emotion. The HRV is a non-invasive and reliable method to assess the activity of ANS. By monitoring the change in HRV, it is possible to analyze the ANS condition during archery contest. Based on previous literature, we could conclude that the activation of parasympathetic nervous system is the key point in improving archery performance. It is important to find an intervention that is easy to perform, less irritating, and non-pharmacological (no risk of doping). Traditional Chinese medicine has been incorporated in sports medicine in recent years. Acupuncture focusses on the characters of treating from distant parts and regulating the entire body function. Ear acupressure possesses the advantages of being convenient, immediate, non-invasive, and less irritative.

How acupuncture affect the ANS and HRV had been discussed in a systematic review by Lee et al.^[15]^ Publications until October 2009 had been searched on 14 databases without language restrictions. Twelve randomized control trials met the inclusion criteria and had been included. The risk of bias in each study was assessed using the Cochrane criteria. Five randomized control trials revealed significant differences in HRV between patients treated with acupuncture compared with those with sham acupuncture (control group). However, most other randomized controlled trials showed inconsistent results or no significant HRV differences between subjects with acupuncture treatment and sham acupuncture. The review concluded that sham-controlled randomized controlled trials showed variable results and no clear evidence about the specific effect of acupuncture on HRV. Further research is imperative.

Non-invasive auricular stimulation (ear acupressure) was found to affect both meridian activities and HRV in middle-aged women in weight reduction.^[16]^ In post-caesarean section women, those who received ear acupressure had significantly lower heart rate, and anxiety and fatigue symptoms than women with usual care at 5 days postpartum.^[17]^ In a study involving 14 healthy volunteers, significant heart rate decrease and HRV total increase were observed during ear acupressure and/or ear acupressure vibration over auricular heart point.^[18]^

In this study protocol, we will perform ear acupressure with Vaccaria seeds and sham acupressure in both groups of participants in a crossover design. The preceding test had confirmed the similarity of Vaccaria seeds and black mark on 3 M tape to assure the completion of blinding. The immersive course and the setting protocol of this experiment could mimic the actual change of mental and physical status during competitions. The outcome evaluation will include change of meridian activities and balance index with Ryodoraku device, HRV with continuous heart rate recording, motion stability with WIMU, attention and fatigue levels with self-reported questionnaires, and the scores of the 2 sections as the performance. In conclusion, we hypothesized that ear acupressure could cause immediate effect of modulating the ANS and meridian activities, and consequently improve archery performance.

## Acknowledgments

The authors would like to extend their appreciation to all the archers who will participate in this study, and Feng-Xi junior high school and National Sun Yat-sen University for providing the standard playing field. They appreciate the assistance with the statistical analysis provided by the Biostatistics Center, Kaohsiung Chang Gung Memorial Hospital.

## Author contributions

**Conceptualization:** Szu-Ying Wu, Chun-En Aurea Kuo.

**Data curation:** Szu-Ying Wu, Yun-Ru Lai, Yu Tsao.

**Formal analysis:** Yun-Ru Lai, Yu Tsao.

**Investigation:** Yi-Hsun Tsai.

**Methodology:** Szu-Ying Wu, Wen-Long Hu, Cheng-Hsien Lin.

**Project administration:** Yi-Hsun Tsai, Yu Tsao, Chun-En Aurea Kuo.

**Supervision:** Wen-Long Hu, Ke-Tien Yen, Cheng-Hsien Lin.

**Validation:** Cheng-Hsien Lin.

**Visualization:** Ke-Tien Yen.

**Writing – original draft:** Yi-Hsun Tsai, Chun-En Aurea Kuo.

**Writing – review & editing:** Chun-En Aurea Kuo.
